# Correlations among fatigue, respiratory function, balance and core muscle morphology in multiple sclerosis: a comprehensive observational study

**DOI:** 10.3389/fneur.2024.1529057

**Published:** 2025-01-20

**Authors:** Marta De La Plaza San Frutos, Ismael Sanz-Esteban, Federico Salniccia, Alberto Bermejo-Franco, Ameyalli García-Corona, María Cristina Palma-Baquedano, Marina Castel-Sánchez, Cecilia Estrada-Barranco

**Affiliations:** ^1^Department of Physiotherapy, Faculty of Medicine, Health and Sports, European University of Madrid, Madrid, Spain; ^2^Neurosciences and Physical Therapy Research Group, Madrid, Spain

**Keywords:** multiple sclerosis, fatigue, respiratory function, balance, ultrasound, abdominal muscles

## Abstract

**Background:**

Recent scientific interest has focused on exploring the potential relationships between fatigue, respiratory function and balance in multiple sclerosis (MS) subjects. While some studies suggest that fatigue may impact respiratory function and postural stability, the exact nature of these associations remains under investigation. Ultrasound imaging is increasingly being used to examine the structural and functional characteristics of core muscles, aiming to better understand how these variables might be interconnected. Understanding these associations is crucial for developing targeted interventions to enhance overall physical performance in this population. This study examines the relationship between fatigue, respiratory function, balance, and ultrasound variables of abdominal musculature in MS.

**Methods:**

A cross-sectional study was conducted involving 27 subjects diagnosed with MS, comprising 17 females and 10 males. Study variables were: fatigue (Modified Fatigue Impact Scale—MFIS); respiratory function (Forced Vital Capacity—FVC and Forced Expiratory Volume in the first second—FEV1); balance (Berg Balance Scale—BBS and Trunk Impairment Scale Dynamic—TIS DYN); and ultrasound measurements of abdominal and diaphragmatic musculature. Correlations were analyzed using Spearman’s correlation, with a statistical significance level of *p* < 0.05.

**Results:**

Significant correlations were found between respiratory function and balance scores. FVC showed a moderate correlation with BBS (*r* = 0.443), while FEV1 had a high correlation with BBS (*r* = 0.500) and a moderate correlation with TIS DYN (*r* = 0.427). MFIS showed a moderate negative correlation with BBS (*r* = −0.402). The strength and function of the central abdominal musculature, particularly the internal oblique, were crucial for trunk stability and postural control.

**Conclusion:**

These findings highlight the interplay between respiratory function, balance, fatigue, and abdominal muscle morphology in MS, emphasizing the potential benefits of interventions targeting respiratory function to improve balance and reduce fatigue, ultimately enhancing quality of life in this population.

## 1 Introduction

Multiple sclerosis (MS) is a chronic disease of the central nervous system (CNS) characterized by inflammation, demyelination, and axonal degeneration, primarily affecting young adults (1). The symptoms vary among patients, with the principal manifestations ranging from fatigue and impairments in balance and respiratory function to cognitive and mobility alterations (2, 3).

Subjects with MS frequently experience fatigue, which can be categorized into two types: central fatigue, resulting from central nervous system alterations, and peripheral fatigue, associated with changes in neuromuscular function (4–6). Studies suggest a potential link between fatigue and nervous system structural changes, including cortico-subcortical grey matter atrophy and basal ganglia circuit impairment, accompanied by neurophysiological decrements, which include reduced strength and voluntary activation (4, 7). Additionally, prior research has established a connection between fatigue variables and balance systems and postural control in MS subjects (8–11).

Due to CNS damage, MS individuals experience impaired respiratory function, resulting from muscle weakness and trunk postural dysfunction (12). Evidence shows a reduction in respiratory muscle strength and lung volumes, along with the development of ineffective coughing and retention of secretions, which increases morbidity and mortality from respiratory problems (13). Inspiratory muscle training is an effective therapeutic strategy to strengthen the respiratory muscles. This training has demonstrated numerous benefits, such as increased inspiratory muscle strength and endurance, improved lung function, increased exercise capacity, decreased dyspnea and reduced risk of pulmonary complications (14, 15).

Although research has focused primarily on the benefits of inspiratory muscle training on the cardiorespiratory system, improving respiratory muscle strength may also have some effect on postural control and balance (16, 17). Hodges and Gandevia state that the diaphragm has a stabilizing function and acts both indirectly, by increasing intra-abdominal pressure and supporting the spine, and directly, by continuous co-contraction that contributes to postural stabilization (18). Previous studies have demonstrated the relationship between postural control and respiratory function in other pathologies, such as stroke patients, Parkinson’s disease and healthy subjects (19–21). Despite the significant impact of MS on both postural stability and respiratory function, there is a notable gap in the literature addressing this relationship within the MS population (5, 22). Good postural control improves respiratory capacities, and conversely, optimal respiratory function enhances postural control (21). On the other hand, if postural control is compromised, the diaphragm may not participate as effectively in ventilatory mechanics (8).

Previous research reported a positive correlation between fatigue and decreased respiratory capacities in MS population (23, 24). Similarly, other authors identified a correlation between respiratory function training and an improvement in perceived fatigue in MS (25, 26). A recent study found that subjects with MS experiencing reduced postural stability and balance also showed impaired respiratory function and fatigue symptoms (22). However, the study noted that the observed respiratory limitations and fatigue were not directly correlated with each other.

People with MS frequently present alterations in the core muscle complex. Concretely, the term “core” has been widely used in order to refer to a belt-like tension to the trunk provided by the automatic activation of deep muscles like the transversus abdominis and internal oblique, multifidus, diaphragm and pelvic floor (27). One component of balance is postural stability of the trunk; it is commonly referred to as “core stability” (18). Core stability is defined as the motor control and muscular ability in the abdominal area to remain stable in different postures and with external forces (28). In a patient with MS, the stability of this area is affected by three closely interacting and related systems, which are: the passive bone and ligament systems, the active muscle systems and the nervous system; any impairment in one system is compensated for by the others. Several studies show that the core is directly affected by the pathology and that it is necessary to perform a treatment focused on strengthening the surrounding musculature in order to improve the postural and respiratory pattern (29).

Several valid and reliable tools were used to evaluate static and dynamic muscular conditions, such as electromyography (EMG), magnetic resonance imaging (MRI) and rehabilitative ultrasound imaging (RUSI), which may be considered as a conservative, non-invasive, non-expensive, valid and reliable tool for measuring the thickness at rest and during contraction of the deep trunk muscles (30). Prior research considered relevant to use ultrasonographic evaluation of the diaphragm thickness in MS (31).

The purpose of this research is to provide information on the correlation between fatigue, respiratory function, balance, and ultrasound variables of the abdominal muscles in MS subjects. Despite advances in pharmacological treatments, many aspects of MS, especially those related to the aforementioned variables, remain poorly understood and studied. The therapeutic management of fatigue continues to be a challenge due to its multifactorial nature and the lack of effective treatments specifically targeting this symptom. Additionally, MS directly affects the respiratory muscles, a condition that over time can become a restrictive syndrome, leading to the death of nearly half of MS population (32–34). Therefore, our research aims to enhance scientific understanding and improve therapeutic interventions for the physical and functional aspects of MS population. The primary objective of the present study is to analyze the relationship between respiratory function and balance in MS. Secondary goals are to investigate the relationship between fatigue and respiratory function, and the relationship among fatigue, respiratory function and ultrasound variables of abdominal muscles in MS.

## 2 Materials and methods

### 2.1 Study design

An observational, cross-sectional, analytical study was conducted on fatigue, respiratory function, balance and ultrasound variables of the abdominal musculature in people with MS, following the STROBE (Strengthening the Reporting of Observational Studies in Epidemiology) statement for observational studies (35).

This study was approved by the Ethics Committee of the Hospital Clínico San Carlos with code (C.I. 23/685-E).

### 2.2 Setting

All measurements (pulmonary function, balance and ultrasound variables) were collected in a single session in the MS Foundation of Madrid (FEMM). The evaluations were conducted in the following sequence: Trunk Impairment Scale (TIS) assessment, ultrasound measurements, Berg Balance Scale (BBS) assessment, and spirometry measurements. To ensure accuracy and minimize fatigue, participants were given a 5-min rest period after the TIS assessment, a 2-min rest between each ultrasound measurement, a 5-min rest after the BBS assessment, and a 5-min rest between each spirometry maneuver. This structured approach was designed to maintain consistency and reliability in the results.

### 2.3 Study size

The sample size was calculated using GRANMO© software (version 8.0), accepting an alpha risk of 0.05 and a statistical power of 80% in a bilateral contrast. Assuming a correlation coefficient of 0.5, the required sample size was determined to be 30 participants.

### 2.4 Participants

Participants were recruited from the FEMM during March to May 2024. This was facilitated through a previously established agreement with the Madrid Foundation Against Multiple Sclerosis, the Official College of Pharmacists of Madrid, the Real Madrid Foundation, Sanitas, and the European University of Madrid. All participants provide written informed consent.

### 2.5 Eligibility criteria

The inclusion criteria for this sample were people diagnosed with confirmed diagnosis of MS based on the McDonald criteria, with a disease duration of more than 2 years (relapsing–remitting or progressive) (36, 37), aged 18 to 70 years old, stable medical treatment for at least 6 months prior to intervention (38), absence of cognitive impairment, with the ability to understand instructions and achieve a score of 24 or higher on the Mini-Mental State Examination (39), with a score less than or equal to 6 on the Kurtzke Expanded Disability Status Scale (EDSS) (40). Participants were included regardless of wheelchair use. Walking-related test were only given to those who could walk.

Exclusion criteria were subjects with a diagnosis of another neurological disease or musculoskeletal disorder, a diagnosis of any cardiovascular, respiratory, or metabolic disease, or other conditions that could interfere with this study, those who have experienced an exacerbation or hospitalization in the 3 months prior to the assessment protocol or during the therapeutic intervention process, and those who have received a cycle of steroids, either intravenously or orally, 6 months before the start of the assessment protocol and during the study intervention period.

### 2.6 Variables and measurement

We collected the variables age, weight and height from each participant, considered crucial for comparative analysis with reference measures in spirometry, maximum inspiratory pressure, and maximum expiratory pressure.

#### 2.6.1 Respiratory function

The functional spirometry test was used for measurement, following the SEPAR Normative criteria (2013) (41). An open-system pneumotachograph or spirometer was used (Easy on-PC Spirometer®; ndd Medizintechnik AG). Forced Vital Capacity (FVC), the maximum volume of air in milliliters that the participant can inhale during a forced inspiration maneuver, was obtained. Additionally, the Forced Expiratory Volume in the first second (FEV1), measured in milliliters, was recorded to provide information on pulmonary elastic quality. A minimum of 3 maneuvers and a maximum of 8 were performed, from which the best values for FVC and FEV1 were recorded, even if they were from different maneuvers (42).

Respiratory muscle strength was determined by the variables maximal inspiratory pressure (MIP) and maximal expiratory pressure (MEP). The highest value of three repetitions of MIP and the highest value of MEP (cm^3^ H₂O), were taken and compared with reference values (42).

#### 2.6.2 Core muscle morphology

The morphological evaluation of diaphragm and abdominal wall muscles was assessed by ultrasound measurements, using the LOGIQ S7 Expert® ultrasound machine from General Electric. To avoid biases and in accordance with RUSI (Rehabilitative Ultrasound Imaging) guidelines, three measurements were taken for each examination to record the average of the three (43). For the assessment of diaphragmatic thickness, we utilize a linear probe with a frequency range of 10–12 MHz. Participants were comfortably positioned in the supine posture, and the examination focuses on the anterior aspect of the right hemidiaphragm. Using the hepatic window as our point of access, the ultrasound probe is positioned perpendicularly within the intercostal space between the ninth and tenth ribs, aligned with the axillary line (44). Within the ultrasound image, we identify three distinct parallel layers, each with varying echogenicity corresponding to the pleura, diaphragm, and peritoneum. To measure diaphragmatic thickness, we employ the M-mode, freezing the image during an unforced expiration. Three separate measurements are taken during each examination. and the final result is derived from the mean of these three measurements.

For the assessment of diaphragmatic excursion, we use a convex ultrasound probe with a frequency range of 2.5 to 3.5 MHz. Participants were placed in the supine position with a 45° headrest angle and are encouraged to rest quietly with closed eyes before the examination. The ultrasound probe is positioned beneath the right costal arch, along a line corresponding to the midpoint of the clavicle and oriented cranially. This positioning allows us to visualize the dome of the right hemidiaphragm as a prominent hyperechoic line. To measure diaphragmatic excursion, we record measurements for both normal and forced breathing. Conducting this process three times, the measurement is taken from the highest point of the sinuous curve of the diaphragmatic dome to the lowest point it reaches during inspiratory contraction (45). The final result is calculated as the mean of the three measurements. Standardization of these procedures guarantees consistency in measurement and results across our study (45, 46).

For evaluating abdominal diastasis, the participant was positioned lying on their back with knees bent at 90° and arms along the body (47). The transducer was placed transversely along the midline of the abdomen, 2 cm below the center of the navel, with the measurement marked on the skin beforehand. Images were captured immediately at the end of exhalation (47). Three measurements were taken at the same point for each participant to calculate the average.

For the ultrasound measurement of the abdominal wall, the linear probe was positioned at the level of the navel and on the anterolateral part of the abdomen. The muscle layers of the transverse, internal oblique, and external oblique appeared. The cross-sectional thickness of the three muscles was measured, taking the anterior insertion point of the transverse muscle as a reference. Measurements were taken at the end of a quiet exhalation without forcing and at the end of a forced exhalation (48).

#### 2.6.3 Balance

The Berg Balance Scale (BBS) was used to measure balance through functional abilities in standing. It consists of 14 items, each rated from 0 to 4 (the higher the score, the better the balance), with a total score of 56 points (49).

#### 2.6.4 Trunk control

The Trunk Impairment Scale (TIS) was used to assess motor impairment of the trunk. It evaluates three main aspects: static sitting balance (TIS STA), dynamic sitting balance (TIS DYN), and trunk coordination (TIS COO). It consists of 17 items with scores ranging from 0 to 23. A higher score indicates better balance. This scale is validated for multiple sclerosis (50).

#### 2.6.5 Fatigue

The Modified Fatigue Impact Scale (MFIS) is a self-assessment questionnaire that measures the impact of fatigue on physical, cognitive, and psychosocial functions in MS. It consists of 21 items rated on a 4-point scale. The total MFIS score can range from 0 to 84, calculated by summing the scores of the physical, cognitive, and psychosocial subscales (51–54).

### 2.7 Statistical methods

Statistical analysis included Spearman correlation to explore relationships between respiratory function, core muscles ultrasound variables, balance and fatigue. According to Hopkins et al. (55), the following levels of correlation coefficient were established: non-existent (*r* < 0.1); low (*r* = ≥0.1 < 0.3); moderate (*r* = ≥0.3 < 0.5); high (*r* = ≥0.5 < 0.7); very high (*r* = ≥0.7 < 0.9) and almost perfect (*r* ≥ 0.9). A statistical significance level of *p* = 0.05 was set.

## 3 Results

A total of 30 participants were recruited for evaluation, of which 3 were excluded for not meeting the inclusion criteria: one for not having a clear diagnosis and two for having comorbidities that made them susceptible to exclusion. The sociodemographic characteristics are shown in Table 1.

**Table 1:** Sociodemographic characteristics of the participants.

Characteristic	Participants (*n* = 27)
Age (years)	47 ± 11.06
Females (%)	17 (62.96%)
Males (%)	10 (37.03%)
Weight (kg)	170.3 ± 8.09
Height (cm)	71.6 ± 13.43
Primary-Progressive MS	5 (18.51%)
Secondary-Progressive MS	2 (7.40%)
Relapsing- Remitting MS	20 (74.07%)

Data are presented in absolute frequency or in median ± standard deviation; MS, Multiple Sclerosis.

Descriptive statistics of our study variables are shown in Table 2.

**Table 2:** Descriptive statistics of the study variables.

Variables	Median (Q1-Q3)	Variables	Median (Q1-Q3)
TIS	13.5 (5.875–21.125)	*TR REST*	0.25 (0.165–0.335)
TIS STA	3.0 (2.500–3.500)	*TR CONT*	0.4 (0.260–0.540)
TIS DYN	7.0 (5.875–8.125)	*IO REST*	0.53 (0.360–0.700)
TIS COO	3.5 (2.500–4.500)	*IO CONT*	0.78 (0.605–0.955)
BBS	42.5 (38.000–47.000)	*EO REST*	0.38 (0.235–0.525)
FVC	90.5 (76.750–104.250)	*EO CONT*	0.43 (0.235–0.625)
FEV1	84.0 (73.125–94.875)	*DX REST*	0.15 (0.120–0.180)
FEV/FVC	78.0 (68.125–87.875)	*DX CONT*	0.29 (0.230–0.350)
MIP	51.0 (32.000–70.000)	*DX EX*	5.91 (4.450–7.370)
MEP	59.5 (40.750–78.250)	*MFIS*	45.0 (38.250–51.750)

TIS, Trunk Impairment Scale; TIS STA, Trunk Impairment Scale Static; TIS DYN, Trunk Impairment Scale Dynamic; TIS COO, Trunk Impairment Scale Coordination; BBS, Berg Balance Scale; MFIS, Modified Fatigue Impact Scale; FVC, Forced Vital Capacity; FEV1, Forced Expiratory Volume in the first second; MIP, maximal inspiratory pressure; MEP, maximal expiratory pressure; TR REST, Thickness of transverse muscle during rest; TR CONT, Thickness of transverse muscle in contraction; IO REST, Thickness of the internal oblique at rest; IO CONT, Thickness of the internal oblique during contraction; EO REST, Thickness of the external oblique at rest; EO CONT, Thickness of the external oblique during contraction; DX REST, diafragmatic thickness at rest; DX CONT, Diafragmatic thickness during the contraction; DX EX, Diafragmatic Excursion during inspiration; Q1, First Quartil; Q3, Third Quartil.

Associations between measures of respiratory function, balance and fatigue in MS subjects are shown in Table 3. The findings indicated that FVC and FEV1 are significantly correlated with BBS, with coefficients of 0.443 and 0.500, respectively. Additionally, FEV1 also showed a moderate correlation with TIS DYN (*r* = 0.427), and the FEV/FVC ratio presented a moderate correlation with TIS DYN (*r* = 0.425). Fatigue, measured by MFIS, had a moderate negative correlation with BBS (*r* = −0.402).

**Table 3:** Correlations between fatigue, balance and respiratory function.

	TIS r (*p*)	TIS STA r (*p*)	TIS DYN r (*p*)	TIS COO r (*p*)	BBS r (*p*)	MFIS r (*p*)
FVC	0.194 (*p* = 0.341)	0.207 (*p* = 0.310)	0.125 (*p* = 0.543)	0.276 (*p* = 0.173)	**0.443 (*p* = 0.023)**	−0.073 (*p* = 0.719)
FEV1	0.344 (*p* = 0.085)	0.146 (*p* = 0.477)	**0.427** (***p* = 0.030**)	0.260 (*p* = 0.2)	**0.5 (*p* = 0.009)**	−0.019 (*p* = 0.104)
FEV1/FVC	0.375 (*p* = 0.059)	0.217 (*p* = 0.287)	**0.425 (*p* = 0.030)**	0.252 (*p* = 0.214)	0.091 (*p* = 0.659)	0.320 (*p* = 0.321)
MIP	0.345 (0.08)	0.319 (*p* = 0.112)	0.226 (*p* = 0.267)	0.344 (*p* = 0.085)	0.199 (*p* = 0.331)	0.000 (*p* = 0.99)
MEP	0.365 (*p* = 0.66)	0.324 (*p* = 107)	0.206 (*p* = 312)	**0.413 (*p* = 0.036)**	0.286 (*p* = 0.157)	−0.028 (*p* = 0.89)
MFIS	−0.2 (*p* = 0.328)	−0.275 (*p* = 174)	−0.206 (*p* = 313)	−0.098 (*p* = 0.63)	**−0.402 (*p* = 0.04)**	
BBS	***0.553* (p = 0.003)**	***0,422 (p = 0.032)***	***0,545 (p = 0.004)***	***0,474 (p = 0.015)***		

TIS, Trunk Impairment Scale; TIS STA, Trunk Impairment Scale Static; TIS DYN, Trunk Impairment Scale Dynamic; TIS COO, Trunk Impairment Scale Coordination; BBS, Berg Balance Scale; MFIS, Modified Fatigue Impact Scale; FVC, Forced Vital Capacity; FEV1, Forced Expiratory Volume in the first second; MIP, Maximal Inspiratory Pressure; MEP, Maximal Expiratory Pressure.Bold type in tables indicates statistical significance.

**Figure 1:**
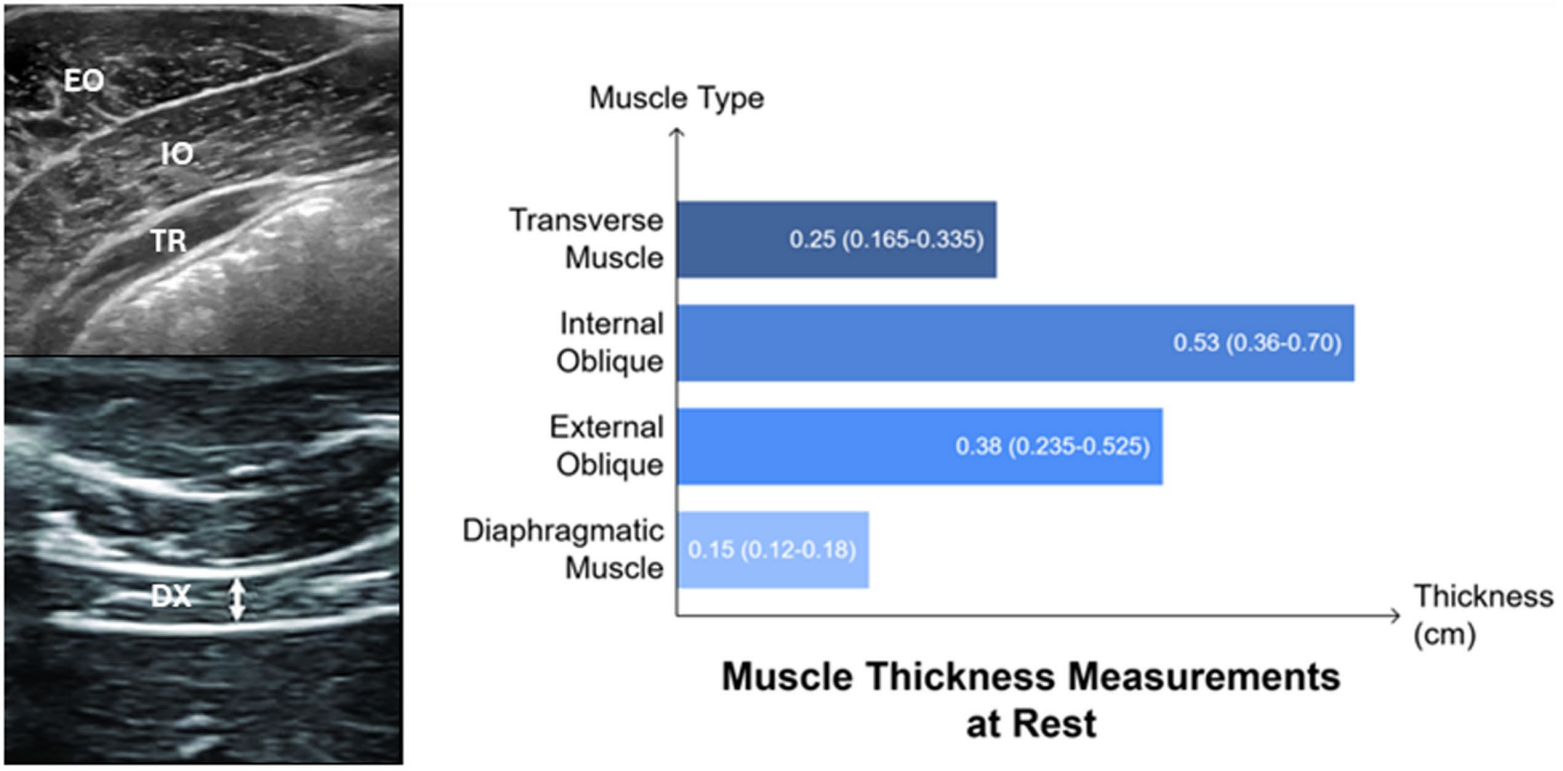
Resting measurements of abdominal and diaphragmatic muscle thickness. EO, external oblique; IO, internal oblique; TR, transverse; DX, diaphragm.

**Figure 2:**
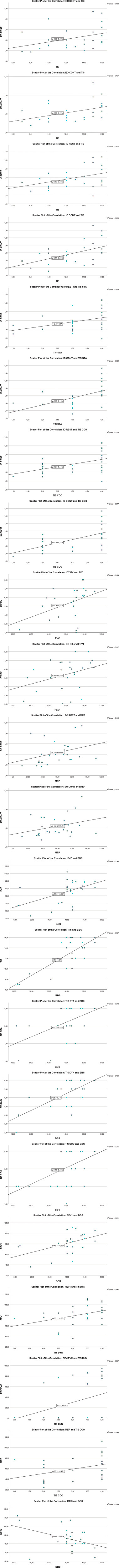
Scatter plots of significant correlations between ultrasound measurements, respiratory function, balance, and fatigue in MS.

Table 4 illustrated the correlations between the thickness of abdominal and diaphragmatic muscles, as well as diaphragmatic excursion, with balance, fatigue, and respiratory function variables. Figure 1 showed resting measurements of abdominal and diaphragmatic muscle thickness. The results indicated that the thickness of the internal oblique during contraction had a high correlation with TIS STA (*r* = 0.655, *p* < 0.001) and TIS COO (*r* = 0.641, *p* < 0.001), and a moderate correlation with TIS DYN (*r* = 0.551, *p* = 0.004). Additionally, diafragmatic excursion during inspiration showed a moderate correlation with FVC (*r* = 0.489, *p* = 0.010) and FEV1 (*r* = 0.333, *p* = 0.090). On the other hand, fatigue measured by MFIS presents a high negative correlation with diafragmatic thickness at rest (*r* = −0.629, *p* < 0.001).Figure 2 showed scatter plots of significant correlations between ultrasound measurements, respiratory function, balance, and fatigue in MS.

**Table 4:** Correlations between the thickness of abdominal muscles, diaphragm, and diaphragmatic excursion and balance, fatigue, and respiratory function variables.

	TR REST r (*p*)	TR CONT r (*p*)	EO REST r (*p*)	EO CONT r (*p*)	IO REST r (*p*)	IO CONT r (*p*)	DX REST r (*p*)	DX CONT r (*p*)	DX EX r (*p*)
TIS	0.244 (*p* = 0.23)	−0.14 (*p* = 0.47)	**0.43 (*p* = 0.028)**	**0.487 (*p* = 0.01)**	**0.386 (*p* = 0.05)**	**0.551 (*p* = 0.004)**	−0.018 (*p* = 0.931)	0.114 (*p* = 0.58)	0.071 (*p* = 0.732)
TIS STA	0.153 (*p* = 0.454)	−0.08 (*p* = 0.67)	0.26 (*p* = 0.2)	0.348 (*p* = 0.081)	**0.385 (*p* = 0.05)**	**0.655 (*p* < 0.001)**	−0.009 (*p* = 0.967)	0.184 (*p* = 0.369)	0.041 (*p* = 0.84)
TIS DYN	0.245 (*p* = 0.22 8)	−0.18 (*p* = 0.361)	0.326 (*p* = 0.104)	0.377 (*p* = 0.058**)**	0.218 (*p* = 0.28)	0.229 (*p* = 0.26)	0.110 (*p* = 0.594)	0.106 (*p* = 0.606)	0.157 (*p* = 0.44)
TIS COO	0.99 (*p* = 0.63)	−0.182 (*p* = 0.37)	0.362 (*p* = 0.069)	0.491 (*p* = 0.13)	**0.459 (*p* = 0.018)**	**0.641 (*p* < 0.001)**	−0.133 (*p* = 0.517)	0.176 (*p* = 0.390)	0.081 (*p* = 0.69)
BBS	0.204 (*p* = 0.32)	0.147 (*p* = 0.47)	0.218 (*p* = 0.28)	0.262 (*p* = 0.19)	0.054 (*p* = 0.793)	0.175 (*p* = 0.39)	0.253 (*p* = 0.213)	0.089 (*p* = 0.666)	0.233 (*p* = 0.252)
FVC	−0.115 (*p* = 0.57)	−0.181 (*p* = 0.36)	0.121 (*p* = 0.54)	0.116 (*p* = 0.56)	0.055 (*p* = 0.786)	−0.07 (*p* = 0.73)	0.213 (*p* = 0.285)	−0.066 (*p* = 0.742)	**0.489 (*p* = 0.01)**
FEV1	−0.019 (*p* = 0.92)	−0.292 (*p* = −0.14)	0.059 (*p* = 0.77)	0.21 (*p* = 0.917)	−0.17(*p* = 0.397)	−0.246 (*p* = 0.217)	0.257 (*p* = 0.195)	−0.046 (*p* = 0.821)	**0.333 (*p* = 0.09)**
FEV1/FVC	0.275 (*p* = 0.16)	−0.96 (*p* = 0.63)	−0.095 (*p* = 0.63)	0.162 (*p* = 0.42)	0.15 (*p* = 0.939)	−0.027 (*p* = 0.89)	0.033 (*p* = 0.87)	0.102 (*p* = 0.614)	−0.043 (*p* = 0.833)
MIP	0.073 (*p* = 0.71)	0.106 (*p* = 0.599)	0.32 (*p* = 0.1)	0.295 (*p* = 0.136)	0.194 (*p* = 0.33)	0.326 (*p* = 0.09)	−0.237 (*p* = 0.235)	−0.307 (*p* = 0.119)	−0.081 (*p* = 0.687)
MEP	−0.065 (*p* = 0.74)	0.09 (*p* = 0.64)	**0.454 (*p* = 0.017)**	**0.355 (*p* = 0.07)**	0.119 (*p* = 0.556)	0.27 (*p* = 0.16)	−0.14 (*p* = 0.487)	−0.376 (*p* = 0.05)	−0.072 (*p* = 0.723)
MFIS	−0.326 (*p* = 0.09)	−0.023 (*p* = 0.9)	−0.39 (*p* = 0.847)	−132 (*p* = 0.512)	−0.06 (*p* = 0.767)	−0.085 (*p* = 0.67)	−0.629 (*p* < 0.001)	−0.345 (*p* = 0.078)	−0.032 (*p* = 0.875)

TIS, Trunk Impairment Scale; TIS STA, Trunk Impairment Scale Static; TIS DYN, Trunk Impairment Scale Dynamic; TIS COO, Trunk Impairment Scale Coordination; BBS, Berg Balance Scale; FVC, Forced Vital Capacity; FEV1, Forced Expiratory Volume in the first second; MIP, Maximal Inspiratory Pressure; MEP, Maximal Expiratory Pressure; MFIS, Modified Fatigue Impact Scale; TR REST, Thickness of transverse muscle during rest; TR CONT, Thickness of transverse muscle in contraction; EO REST, Thickness of the external oblique at rest; EO CONT, Thickness of the external oblique during contraction; IO REST, Thickness of the internal oblique at rest; IO CONT, Thickness of the internal oblique during contraction; DX REST, diafragmatic thickness at rest; DX CONT, Diafragmatic thickness during the contraction; DX EX, Diafragmatic Excursion during inspiration.Bold type in tables indicates statistical significance.

## 4 Discussion

In this observational study involving subjects with MS, we explored the relationship between fatigue, respiratory function, balance, and ultrasound measurements of abdominal and diaphragmatic musculature. Our findings revealed significant correlations between respiratory function and balance. Specifically, we found that FVC moderately correlated with BBS, while FEV1 showed a moderate correlation with TIS DYN and a high correlation with the BBS. Additionally, fatigue and MEP showed moderate correlations with balance scales and ultrasound measures of abdominal musculature, highlighting the complexity of interactions between balance, respiratory function, and fatigue in MS.

### 4.1 Relationship between respiratory function and balance in MS subjects

Our data analysis reveals a significant correlation between respiratory function and balance scale scores. FVC showed a moderate correlation with the BBS score, while FEV1 exhibited a moderate correlation with the TIS DYN and a high correlation with the BBS. These findings suggest that respiratory function may influence balance capacity in MS. Respiratory muscle weakness and trunk dysfunction can affect postural balance, as indicated by previous studies (32, 55). These results underscore the importance of evaluating and treating respiratory function in MSsubjects, not only to improve respiratory capacity but also to enhance balance and reduce fall risk.

In MS, respiratory muscle weakness is common due to the involvement of neuromuscular pathways controlling these muscles. This can lead to a decrease in FVC and FEV1. The reduction in these capacities not only compromises respiratory function but also negatively impacts postural stability and balance. This is because respiratory muscles play a crucial role in maintaining posture and trunk stability, as mentioned by previous research (3, 20, 56, 57).

### 4.2 Relationship between fatigue and balance

Fatigue, measured by MFIS, showed a moderate correlation with the BBS (Table 3). This result aligns with existing literature, which suggests that fatigue is one of the most common symptoms in MS and can significantly impact functional capacity, including balance and postural stability (58–60). Fatigue in MS can be multifactorial, including muscle weakness, neuromuscular dysfunction, and chronic inflammation (61–63). Fatigue not only reduces the ability to perform daily activities but also affects balance and increases the risk of falls (64, 65).

The relationship between fatigue and balance can be explained by several mechanisms. Fatigue can reduce muscle strength and endurance, which in turn can compromise the ability to maintain a stable posture and respond adequately to balance disturbances. Additionally, fatigue can affect cognitive function and motor coordination, making it difficult to perform tasks requiring precise postural control (66–68).

### 4.3 Ultrasound measurement of abdominal and diaphragmatic musculature

Ultrasound measurements of abdominal and diaphragmatic muscle thickness also revealed significant correlations with balance and respiratory function variables. The thickness of the internal oblique muscle during contraction (OI CONT) showed a high correlation with the TIS STA and TIS COOR, and a moderate correlation with the TIS DYN. These findings suggest that the strength and function of central abdominal musculature, particularly the internal oblique, play a crucial role in trunk stability and postural control in MS, as mentioned in the articles by Mangum et al. and Whittaker & Stokes (43, 48).

Ultrasound measurement of abdominal and diaphragmatic muscle thickness provides valuable information about the status of these muscles in MS. Weakness of these muscles can contribute to postural instability and imbalance (27). Strengthening abdominal muscles in MS patients may enhance respiratory and balance functions. The use of ultrasound to evaluate these muscles allows for precise and objective assessment, which can guide therapeutic interventions (48).

The study by Sánchez-Ruiz et al. (22) explores the relationship between respiratory function, balance, and fatigue in people with MS, and their findings are consistent with the results of our study. In the aforementioned article, it was found that better respiratory function is associated with better postural stability and less fatigue, supporting the hypothesis that interventions aimed at improving respiratory muscle strength can have beneficial effects on balance and fatigue reduction in MS subjects. This parallel between both studies underscores the importance of addressing respiratory function as an integral component in the management of MS to improve quality of life (22). It also highlights the interrelationship between different body systems in MS. Improving respiratory function not only has direct benefits on pulmonary capacity but also positively influences postural stability and fatigue reduction. This suggests that multidisciplinary interventions addressing both respiratory function and balance may be particularly effective in managing MS (55, 69, 70).

### 4.4 Limitations and future research directions

This study has several limitations that should be acknowledged.

Firstly, a potential drawback of using ultrasound for measuring the anatomy of abdominal muscles is the difficulty in differentiating between the external and internal oblique muscles. While this limitation is common across various imaging methods, it is important to mention it for the benefit of readers who may not be familiar with these nuances.

Secondly, the study did not include measurements of the rectus abdominis muscle. Although it is not a deep muscle, the rectus abdominis can play a significant role in expiration, and its exclusion may limit the comprehensiveness of our findings regarding respiratory function.

Additionally, the study did not account for other variables that might explain fatigue at the level of the nervous system. Fatigue in MS is multifactorial, and including additional variables related to nervous system function could provide a more holistic understanding of fatigue mechanisms.

Moreover, the study involved assessing numerous correlations, which raises the potential issue of multiple comparisons. This could increase the risk of Type I errors, where some of the observed correlations might be due to chance rather than representing true associations.

Finally, the cross-sectional nature of the study limits our ability to establish causal relationships. Future longitudinal studies are needed to confirm these findings and to evaluate the effectiveness of specific interventions aimed at improving respiratory function and balance in MS.

## 5 Conclusion

This study highlights the significant correlations between respiratory function, balance, and fatigue in MS. Our findings indicate that FVC and FEV1 are positively correlated with balance scores, suggesting that better respiratory function is associated with improved balance. Additionally, the negative correlation between fatigue (measured by MFIS) and balance underscores the impact of fatigue on postural stability in MS.

The use of ultrasound to assess abdominal and diaphragmatic muscle morphology provided valuable insights into the role of core muscle strength in maintaining balance and respiratory function. The significant correlations between the thickness of the internal oblique muscle and balance measures further emphasize the importance of core stability in this population.

These results suggest that interventions aimed at improving respiratory function and core muscle strength could potentially enhance balance and reduce fatigue in MS patients, ultimately improving their quality of life.

## Data Availability

The raw data supporting the conclusions of this article will be made available by the authors, without undue reservation.
